# Fatigue Properties of the Ultra-High Strength Steel TM210A

**DOI:** 10.3390/ma10091057

**Published:** 2017-09-09

**Authors:** Guang-qiang Yin, Xia Kang, Gui-ping Zhao

**Affiliations:** 1 State Key Laboratory for Strength and Vibration of Mechanical Structures, School of Aerospace, Xi’an Jiaotong University, Xi’an 710049, China; guangqiang12345@163.com (G.-q.Y.); 18200288310@163.com (X.K.); 2 Institute of Aviation Equipment, Qing’an Group Corporation Limited, Xi’an 710077, China

**Keywords:** fatigue, ultra-high strength steel, TM210A, notch

## Abstract

This paper presents the results of an experiment to investigate the high cycle fatigue properties of the ultra-high strength steel TM210A. A constant amplitude rotating bending fatigue experiment was performed at room temperature at stress ratio R = −1. In order to evaluate the notch effect, the fatigue experiment was carried out upon two sets of specimens, smooth and notched, respectively. In the experiment, the rotating bending fatigue life was tested using the group method, and the rotating bending fatigue limit was tested using the staircase method at 1 × 10^7^ cycles. A double weighted least square method was then used to fit the stress-life (S–N) curve. The S–N curves of the two sets of specimens were obtained and the morphologies of the fractures of the two sets of specimens were observed with scanning electron microscopy (SEM). The results showed that the fatigue limit of the smooth specimen for rotating bending fatigue was 615 MPa; the ratio of the fatigue limit to tensile strength was 0.29, and the cracks initiated at the surface of the smooth specimen; while the fatigue limit of the notched specimen for rotating bending fatigue was 363 MPa, and the cracks initiated at the edge of the notch. The fatigue notch sensitivity index of the ultra-high strength maraging steel TM210A was 0.69.

## 1. Introduction

In order to meet the needs of high weight loads, high-transmission ratio, long life, weight and volume limits of important bearing units such as the leading edge flaps of a certain aircraft, material must have performance qualities such as high strength (σ_b_ ≥ 2058 MPa, σ_0.2_ ≥ 1960 MPa), high plasticity (δ_5_ ≥ 7.5%, ψ ≥ 45%), high toughness (α_k_ ≥ 39.2 J/cm^2^, K_IC_ ≥ 62 MPa·m^1/2^), high hardness (HRC ≥ 51) and high fatigue strength (σ_−1_ ≥ 680 MPa (3 × 10^6^)). In the past many materials have been unable to meet these requirements. Recently, a new ultra-high strength maraging steel TM210A has been developed based on the design of the 18Ni maraging steel. The maraging steels are based on the iron-nickel-cobalt-molybdenum system that has emerged as an outstanding family of materials with exceptional combinations of characteristics. They are applied particulary in automotive, aerospace, nuclear, gear, bearing and other industries [1]. These grades generally contain 18 wt % nickel and are commonly designated as 18Nixxxx, where xxxx is the nominal strength value attained by final heat treatment. Their outstanding attributes include ultra-high strength coupled with high fracture toughness, and excellent formability under hot and cold working conditions. The material is commercially available in different strength ranges from 1400 MPa to 2400 MPa [2]. The materials are low-carbon, and the chemical composition is: C ≤ 0.03%, S ≤ 0.01%, P ≤ 0.01%, Ni18.5%, Co7.5%–9.5%, Mo3.25%–4.8%, Ti ≥ 0.2%, Al≥0.1%, and others B, Zr, Ca [3,4].

At present, the material TM210A has been widely used in multi-type aircraft drive devices, such as gears, shafts, splines and other important bearing structures. These structural parts are unavoidably subjected to alternating loads during aircraft service. Fatigue damage is the main cause of failure of the important structural parts in the aircraft. Statistics show that most of the service failures of aircraft components occur in the form of fatigue damage, which amounts to about 60% of total failures [5].Serious air craft flight accidents involving fatigue-related causes are still occurring at about 100 times per year globally [6].

In this paper, rotating bending fatigue tests were carried out using the ultra-high strength maraging steel TM210Ain order to investigate the fatigue strength of the smooth specimen and the notched specimen, and to observe the fracture morphology. Finally, the fatigue notch sensitivity index was calculated.

## 2. Experiment

### 2.1. Material and Specimen

**Figure 1:**
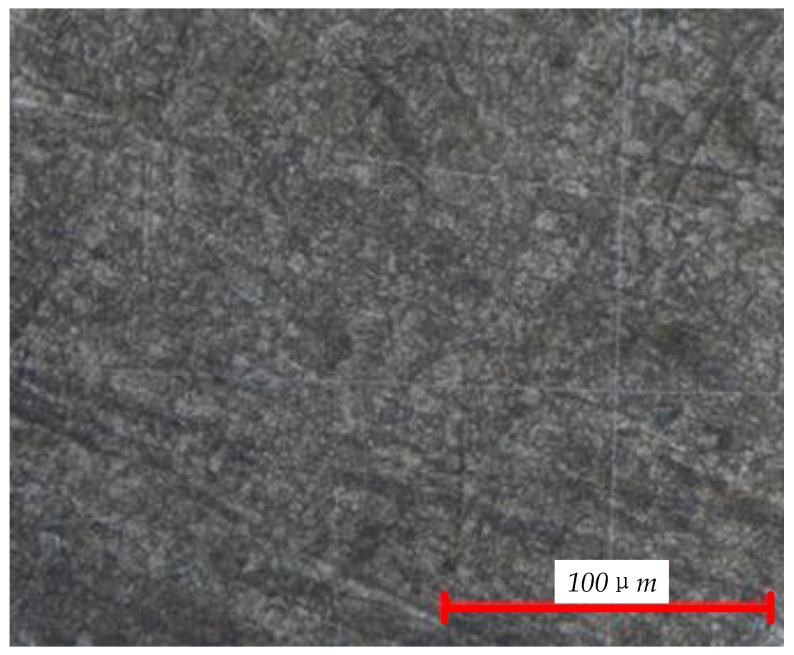
Microstructure of theTM210A.

**Figure 2:**
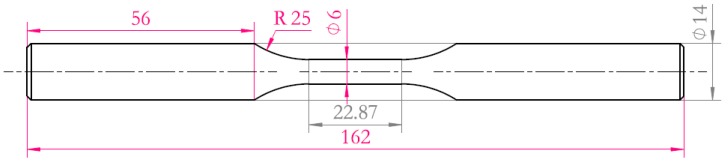
Schematic diagram of smooth round-bar fatigue specimen (unit: mm).

**Figure 3:**
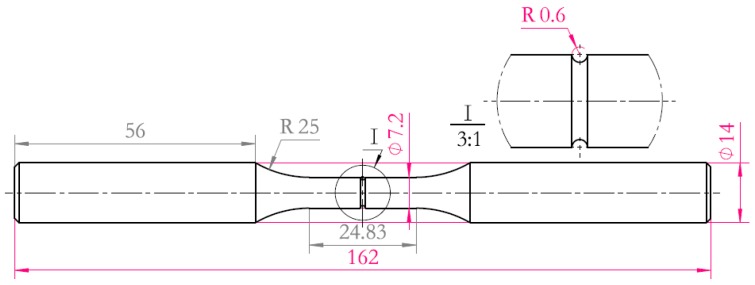
Schematic diagram of notched round-bar fatigue specimen (unit: mm).

The experimental material was ultra-high strength steel TM210A. The chemical composition of TM210A is shown in Table 1. The material was solution-treated for 1h at 820 °C in a vacuum, followed by air-cooling and age-hardening for 4h at 510 ± 5 °C in a salt bath. This method enabled the determination of the plane-strain fracture toughness (K_1C_) by increasing force of fatigue-precracked test specimens. Details of the test specimens and experimental procedures make reference to ISO 12737:2010(E).The mechanical properties of the TM210A are shown in Table 2. The microstructure of TM210A is the martensite shown in Figure 1. The shape and dimensions of the smooth specimen ($K_{t}=1$) and notched specimen ($K_{t}=2$) used in the fatigue experiment are shown in Figure 2 and Figure 3, respectively.

### 2.2. Fatigue Experimental Method

The constant amplitude rotating bending fatigue experimentswere performed in the PQ1-6 bending fatigue experimental machine. The working speed was constant at 5000 r/min. The experimental environment was atmospheric temperature (25 ± 2 °C), humidity (25%–40%RH), and the stress ratio was R = −1. The stress life (S–N) curve of the low/medium cycle fatigue regime was determined using the group method, and the fatigue limit at 1 × 10^7^cycles was determined using the staircase method. The fatigue tests were conducted at various stress levels in order to determine the mean S–N curve with a probability of failure of 50%. The group method of the fatigue properties of metallic engineering materials was determined by testing a set of specimens at various stress levels to generate a fatigue life relationship as a function of stress. The results are expressed as an S–N curve that fits the experimental data, plotted in appropriate coordinates. The fatigue tests were conducted to generate strength data for a set of specimens in a sequential way using the staircase method. This starts the test at a first stress level that is preferably close to the estimated mean strength. It selects a stress step, preferably close to the standard deviation, by which to vary the stress level during the test. If no information is available about the standard deviation, a step of about 5% of the estimated mean fatigue strength may be used as the stress step. A first specimen, randomly chosen, is tested at the first stress level to see if it fails before the given number of cycles. For the next specimen, also randomly chosen, the stress level is increased by a step if the preceding specimen did not fail, and decreased by the same amount if it failed. Testing continues until all the specimens have been tested in this way. The fracture morphology of fatigue specimens was observed using a scanning electron microscope (SEM).

## 3. Results and Discussion

### 3.1. Fatigue Experimental Results

The experiment was carried out from the high stress level to low stress level. The number of cycles to failure, abnormal phenomena, and specimen damage was recorded during the process. The experimental data for smooth and notched specimens of the TM210A are shown in Table 3 and Table 4, respectively. From Table 3 to Table 4, the stress amplitudes of the smooth specimen fatigue test are shown at eight stress levels from 768MPa to 568MPa; and the stress amplitudes of the notched specimen fatigue test at eight stress levels from 473 MPa to 333 MPa. The average fatigue life at each stress level can be calculated, as shown in Table 5 and Table 6.

### 3.2. S–N Curves

The S–N curve is a basic curve for characterizing the fatigue properties of the material. According to the experimental data from Table 3, Table 4, Table 5 and Table 6, the S–N curves of the smooth specimen and the notched specimen can be fitted. The S–N curve has a variety of forms of expression. The three-parameter S–N curve model can be used to describe the stress-life relationship of the long-life region. This study selected the classical three-parameter power function S–N curve model [7].

(1) $$N{\left(S_{\max}-S_{0}\right)}^{H}=C$$

Where $S_{\max}$ is the maximum stress, *N* is the fatigue life, $S_{0}$ is the theoretical fatigue limit, *H* and *C* are the material constant. A double weighted least square method is used for fitting.

From the data in Table 3 and Table 5, the parameters of the smooth specimen were obtained, $S_{0}$ = 613.1649 MPa, H = 0.9665, C = 1.9333 × 10^7^, and the S–N curve equation of the smooth specimen was obtained

(2) $$N{\left(S_{\max}-613.1649\right)}^{0.9665}=1.9333\times 10^{7}$$

**Figure 4:**
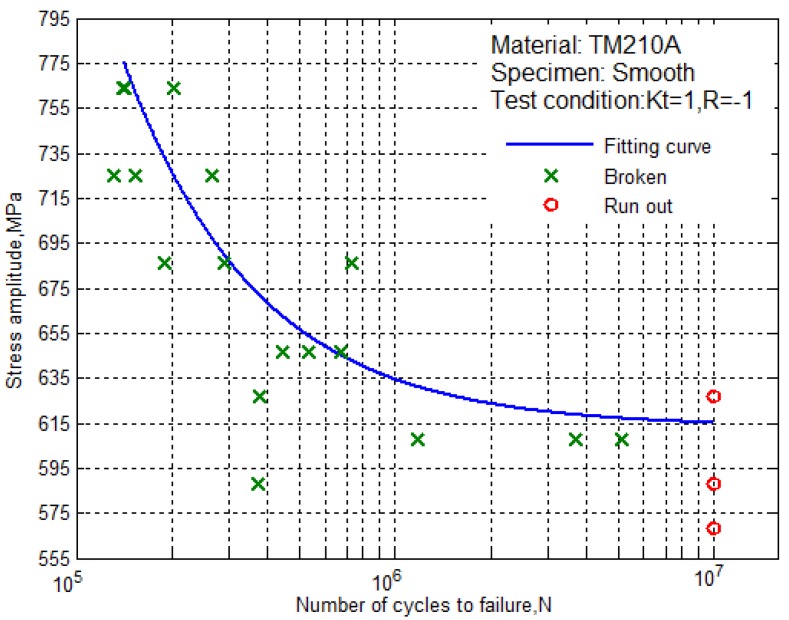
The S–N curve for the smooth specimens with 50% survival rate.

According to the fitting equation, we can draw the S–N curve of the smooth specimens of the ultra-high strength maraging steel TM210A with a 50% survival rate, as shown in Figure 4. It can be seen that the fatigue limit of the smooth specimen was not more than 615 MPa.

From the data in Table 4 and Table 6, the parameters of the notched specimen were obtained, $S_{0}$ = 350.8066MPa, H = 2.0649, C = 1.7488 × 10^7^, and the S–N curve equation of the notched specimen was obtained

(3) $$N{\left(S_{\max}-350.8066\right)}^{2.0649}=1.7488\times 10^{7}$$

**Figure 5:**
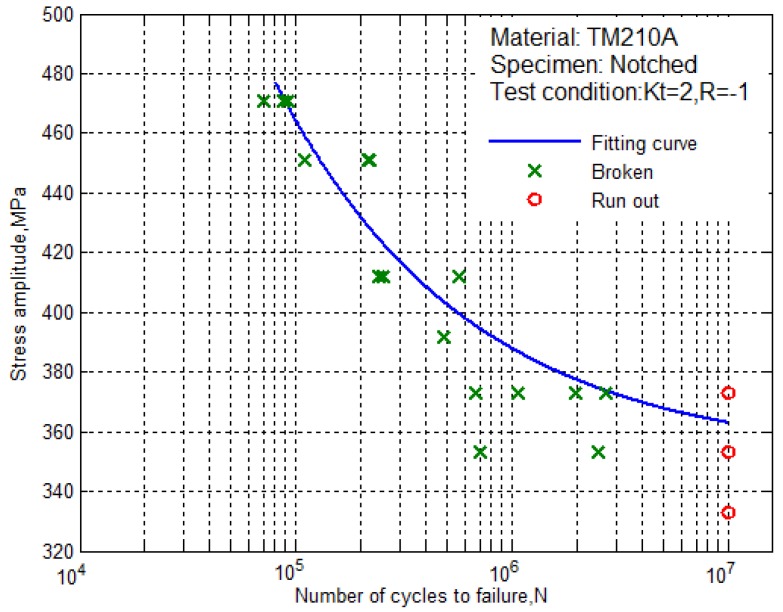
The S–N curve for the notched specimens with 50% survival rate.

According to the fitting equation, we can draw the S–N curve of the notched specimens of the ultra-high strength maraging steel TM210A with a 50% survival rate, as shown in Figure 5. It can be seen that the fatigue limit of the notched specimen was not more than 365 MPa.

From Figure 4 to Figure 5, it can be seen that the S–N curves of the smooth specimens and the notched specimens of the ultra-high strength maraging steel TM210A decreased with the decrease of the stress level, and the fatigue life was prolonged. The fatigue stress-life data contained two parts. Part 1: the data of the test was from initiation to before the fatigue limit, which corresponded to the fracture of the specimen due to the crack initiation-expansion (cross symbols). Part 2: the data of the test was at lower stress levels, in which the specimen did not break (circular symbols).

**Figure 6:**
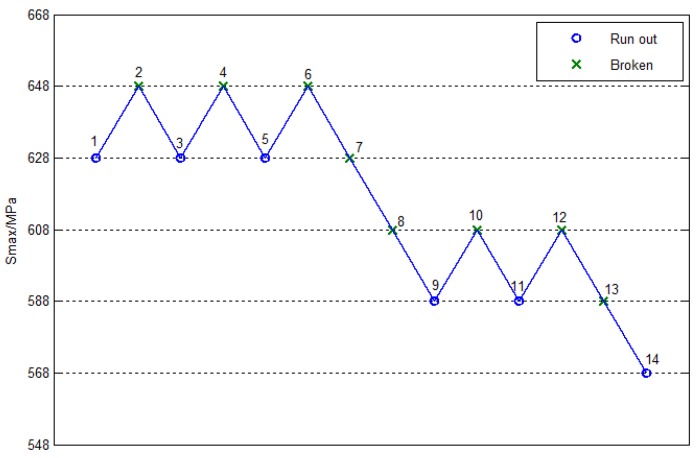
The staircase figure of smooth specimens.

**Figure 7:**
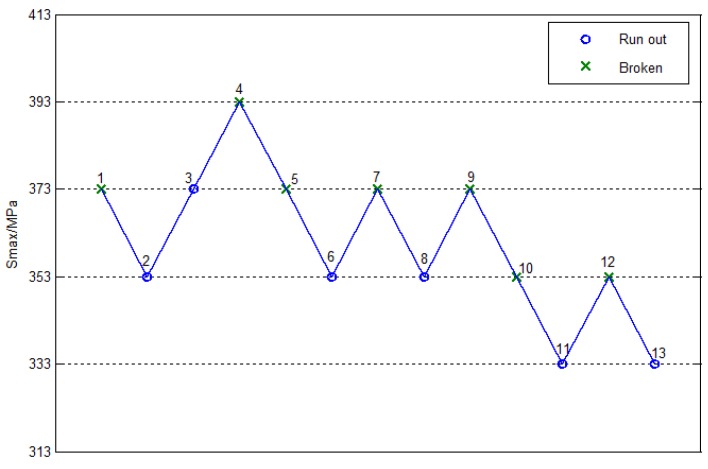
The staircase figure of notched specimens.

For a more intuitive process, Figure 6 and Figure 7 show the fatigue lift graphs for smooth and notched specimens at $1\times 10^{7}$cycles, respectively. Only five stress levels were considered in the analysis of the smooth specimen, as shown in Figure 6, where the beginning stress was 628MPa, the step stress was 20MPa, and the number of the effective specimen was 14. Only four stress levels were considered in the analysis of the notched specimen, as shown in Figure 7, where the stress began at 373 MPa, the step stress was 20 MPa, and the number of the effective specimen was 13.

According to the statistical theory

(4) $$\sigma_{-1}=\frac{1}{k}\sum_{i=1}^{k}\sigma_{i}=\frac{1}{n}\sum_{j=1}^{l}v_{j}\sigma_{j}$$

where $\sigma_{-1}$ is the mean fatigue limit, *k* is the number of matched pairs, *n* is the number of effective specimens, *l* is the number of the stress levels, $\sigma_{i}$ is the *i*-th pair fatigue limit in the matched pair, $\sigma_{j}$ is the *j*-th level stress value of the stress levels, and $v_{j}$ is the number of the effective specimen in the *j*-th level stress level, whether the specimen is broken or not.

Therefore, the fatigue limit of the smooth specimen was 615MPa.The ratio of the fatigue limit to tensile strength was 0.29. The fatigue limit of the notched specimen was 363MPa. Besides this, the fatigue limit of the notched specimen was reduced by about 41% compared with the smooth specimen.

### 3.3. Fractography

**Figure 8:**
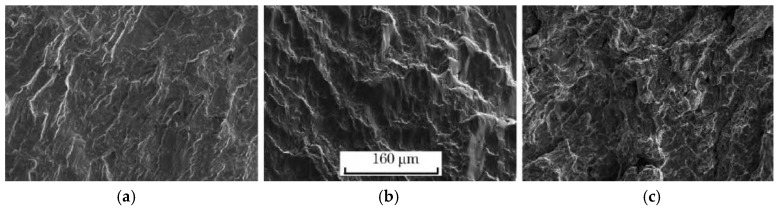
Microstructures of fractures of the smooth specimen: (**a**) Crack initiation; (**b**) Crack propagation; (**c**) Instantaneous fracture

**Figure 9:**
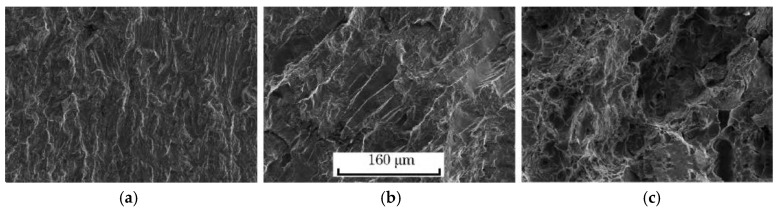
Microstructures of fractures of the notched specimen: (**a**) Crack initiation; (**b**) Crack propagation; (**c**) Instantaneous fracture

The fracture micrographs of the smooth specimens and notched specimens of the ultra-high strength maraging steel TM210A were observed by scanning electron microscopy, as shown in Figure 8 and Figure 9, respectively. It can be seen that the fractures were composed of the crack initiation area, the crack propagation area, and the instantaneous fracture area, whether it was a smooth or notched specimen.

As shown in Figure 8, the crack initiation of the smooth specimen was at the surface of the specimen. In the vicinity of the crack initiation, the crack occurred in a quasi-cleavable way, with a distinct river pattern. The crack propagation area had obvious fatigue striations, and there were some relatively large step shapes. The instantaneous fracture area was a rough dimple.

As shown in Figure 9, the crack initiation of the notched specimen was at the front end of the annular circumference notch with a large stress concentration, in a distinct river pattern. The crack propagation area had obvious fatigue striations, and there were obvious tear edges. The instantaneous fracture area was a rough dimple.

### 3.4. Fatigue Notch Sensitivity Index

The theoretical elastic stress concentration factor *K_t_* relates the local stress ahead of the notch tip to far-field loading, and is defined as the ratio of the maximum local stress σ_max_ to the nominal stress S. Under fatigue loading conditions, the elastic stress concentration factor is replaced by the so-called fatigue notch factor:

(5) $$K_{f}=\frac{\begin{array}{cccc} unnotched & bar & endurance & \lim it \end{array}}{\begin{array}{cccc} notched & bar & endurance & \lim it \end{array}}$$

In general, fatigue experiments suggest that notches produce a less stress-concentrating effect than predicted by theoretical elastic analysis, such that we find *K_f_* ‹ *K_t_; K_f_* → *K_t_* for large notch-root radii and for higher strength materials. The degree of agreement between theoretical predictions of elastic stress concentration and actual effects is often measured by the so-called notch sensitivity index, which is defined as [8]

(6) $$\begin{array}{cc} q_{f}=\frac{K_{f}-1}{K_{t}-1} & \left(0\leq q_{f}\leq 1\right) \end{array}$$

The fatigue limits of the smooth specimen and the notched specimen of the TM210A were 615 MPa and 363 MPa, respectively. The theoretical elastic stress concentration factor *K_t_* was 2, and, therefore, the fatigue notch sensitivity index *q_f_* was 0.69.

## 4. Conclusions

In order to investigate the fatigue strength and the facture mode of the ultra-high strength maraging steel TM210A, rotating bending fatigue tests were carried out using a smooth specimen and a notched specimen. The main results obtained were as follows:

(1) The mean fatigue S–N curve and the mean fatigue limit of the smooth specimen and the notched specimen of the ultra-high strength maraging steel TM210Awere obtained in experiments. The fatigue limits of the smooth specimen and notched specimen were 615 MPa and 363 MPa, respectively. The ratio of the fatigue limit to tensile strength was 0.29. The fatigue limit of the notched specimen was reduced by about 41% compared to the smooth specimen, which indicated that the notch had a great impact on the fatigue properties of materials.
(2) The fracture micrographs of the smooth specimens and notched specimens of the ultra-high strength maraging steel TM210A were composed of a crack initiation area, a crack propagation area, and an instantaneous fracture area. The smooth specimen crack initiated at the specimen surface, and the notched specimen crack initiated at the edge of the notch with a large stress concentration.
(3) The fatigue notch sensitivity index of the ultra-high strength maraging steel TM210A was 0.69.

## Figures and Tables

**Table 1:** Chemical composition of the TM210A (wt %).

C	Si	Mn	S	P	Ni	Co	Mo	Ti	Al
0.003	0.09	0.09	0.005	0.004	17.70	10.32	4.30	0.97	0.10

**Table 2:** Mechanical properties of the TM210A.

Mechanical Property	
Tensile strength σ_b_	2125 MPa
Yield strength σ_0.2_	2065 MPa
Elongation δ_5_	9.5%
Reduction of area ψ	60%
Impact toughness α_k_	42 J/cm^2^
Fracture toughness K_1C_	65 Mpam^1/2^
Hardness	55 HRC

**Table 3:** Fatigue experimental data of the smooth specimens.

Specimen Number	Stress Amplitude	Fatigue Life	Comment	Crack Initiation Point
1	768 MPa	140,200	Broken	At the specimen surface
2	768 MPa	203,200	Broken	At the specimen surface
3	768 MPa	142,100	Broken	At the specimen surface
4	728 MPa	152,700	Broken	At the specimen surface
5	728 MPa	265,300	Broken	At the specimen surface
6	728 MPa	130,500	Broken	At the specimen surface
7	688 MPa	732,300	Broken	At the specimen surface
8	688 MPa	189,400	Broken	At the specimen surface
9	688 MPa	292,300	Broken	At the specimen surface
10	648 MPa	534,300	Broken	At the specimen surface
11	648 MPa	443,000	Broken	At the specimen surface
12	648 MPa	678,600	Broken	At the specimen surface
13	628 MPa	10,077,900	Run out	No
14	628 MPa	10,080,300	Run out	No
15	628 MPa	10,000,000	Run out	No
16	628 MPa	375,300	Broken	At the specimen surface
17	608 MPa	1,174,400	Broken	At the specimen surface
18	608 MPa	5,132,900	Broken	At the specimen surface
19	608 MPa	3,684,100	Broken	At the specimen surface
20	588 MPa	10,000,000	Run out	No
21	588 MPa	10,078,200	Run out	No
22	588 MPa	372,100	Broken	At the specimen surface
23	568 MPa	10,080,600	Run out	No

**Table 4:** Fatigue experimental data of the notched specimens.

Specimen Number	Stress Amplitude	Fatigue Life	Comment	Crack Initiation Point
1	473 MPa	91,300	Broken	At the edge of the notch
2	473 MPa	70,400	Broken	At the edge of the notch
3	473 MPa	86,900	Broken	At the edge of the notch
4	453 MPa	91,300	Broken	At the edge of the notch
5	453 MPa	70,400	Broken	At the edge of the notch
6	453 MPa	86,900	Broken	At the edge of the notch
7	413 MPa	241,600	Broken	At the edge of the notch
8	413 MPa	252,600	Broken	At the edge of the notch
9	413 MPa	563,700	Broken	At the edge of the notch
10	393 MPa	485,500	Broken	At the edge of the notch
11	373 MPa	1,967,700	Broken	At the edge of the notch
12	373 MPa	1,063,000	Broken	At the edge of the notch
13	373 MPa	2,702,000	Broken	At the edge of the notch
14	373 MPa	679,000	Broken	At the edge of the notch
15	373 MPa	10,070,000	Run out	No
16	353 MPa	10,068,700	Run out	No
17	353 MPa	10,068,900	Run out	No
18	353 MPa	10,068,000	Run out	No
19	353 MPa	2,491,300	Broken	At the edge of the notch
20	353 MPa	10,072,700	Run out	No
21	353 MPa	715,300	Broken	At the edge of the notch
22	333 MPa	10,074,000	Run out	No
23	333 MPa	10,074,800	Run out	No

**Table 5:** Fatigue life of the smooth specimens with 50% survival rate.

$S_{\max}$/MPa	768	728	688	615
$N_{50}$/cycle	159,368	174,221	343,558	10^7^

**Table 6:** Fatigue life of the notched specimens with 50% survival rate.

$S_{\max}$/MPa	473	453	413	363
$N_{50}$/cycle	82,357	173,021	325,237	10^7^

## References

[1] Wei S. (2013). Steels.

[2] Rao M.N. (2006). Progress in understanding the metallurgy of 18% nickel maraging steels. Int. J. Mater. Res..

[3] Hoseini S.R.E., Arabi H., Razavizadeh H. (2008). Improvement in mechanical properties of C300 maraging steel by application of VAR process. Vacuum.

[4] Novotny P.M., Maurer G.E. (2007). Ultra-high-strength steels vs. titanium alloys. Adv. Mater. Process..

[5] Bhaumik S.K., Sujata M., Venkataswamy M.A. (2008). Fatigue failure of aircraft components. Eng. Fail. Anal..

[6] Campbell G.S., Lahey R. (1984). A survey of serious aircraft accidents involving fatigue fracture. Int. J. Fatigue.

[7] Little R.E., Jebe E.H. (1975). Statistical Design of Fatigue Experiments.

[8] Suresh S. (1998). Fatigue of Materials.

